# Retraction Note to: Single volar locking plating for the intra- and extra-articular distal radius fractures with dorsal metaphyseal comminution

**DOI:** 10.1186/s13018-022-02962-4

**Published:** 2022-02-09

**Authors:** Xue-yang Gui, Zhao-hui Cheng, Hong-fei Shi, Yi-xin Chen, Jin Xiong, Jun-fei Wang, Xu-sheng Qiu, Zi-tao Zhang

**Affiliations:** 1 Department of Orthopedics, Nanjing Drum Tower Hospital, The Affiliated Hospital of Nanjing University Medical School, No. 321 Zhongshan Road, Nanjing, China; 2 Nanjing Drum Tower Hospital Clinical College of Nanjing Medical University, Nanjing, China

## Retraction Note to: Journal of Orthopaedic Surgery and Research (2021) 16:530 10.1186/s13018-021-02641-w

The authors have retracted this article because after publication it was noted that data from a previous version of the study had been mistakenly included in multiple sections of this article. The authors, therefore, no longer have confidence in the validity of the results. All authors agree to this retraction.

